# A Fully Integrated In Vitro Diagnostic Microsystem for Pathogen Detection Developed Using a “3D Extensible” Microfluidic Design Paradigm

**DOI:** 10.3390/mi10120873

**Published:** 2019-12-12

**Authors:** Zhi Geng, Yin Gu, Shanglin Li, Baobao Lin, Peng Liu

**Affiliations:** 1 Department of Biomedical Engineering, School of Medicine, Tsinghua University, Beijing 100084, China; gengz15@mails.tsinghua.edu.cn (Z.G.); y-gu13@tsinghua.org.cn (Y.G.); li-sl14@tsinghua.org.cn (S.L.); lbb17@mails.tsinghua.edu.cn (B.L.); 2 FengteBio Corporation, Beijing 100079, China

**Keywords:** in vitro diagnostics, microfluidics, full integration, lab-on-a-chip, pathogen detection

## Abstract

Microfluidics is facing critical challenges in the quest of miniaturizing, integrating, and automating in vitro diagnostics, including the increasing complexity of assays, the gap between the macroscale world and the microscale devices, and the diverse throughput demands in various clinical settings. Here, a “3D extensible” microfluidic design paradigm that consists of a set of basic structures and unit operations was developed for constructing any application-specific assay. Four basic structures—check valve (in), check valve (out), double-check valve (in and out), and on–off valve—were designed to mimic basic acts in biochemical assays. By combining these structures linearly, a series of unit operations can be readily formed. We then proposed a “3D extensible” architecture to fulfill the needs of the function integration, the adaptive “world-to-chip” interface, and the adjustable throughput in the X, Y, and Z directions, respectively. To verify this design paradigm, we developed a fully integrated loop-mediated isothermal amplification microsystem that can directly accept swab samples and detect *Chlamydia trachomatis* automatically with a sensitivity one order higher than that of the conventional kit. This demonstration validated the feasibility of using this paradigm to develop integrated and automated microsystems in a less risky and more consistent manner.

## 1. Introduction

Since its inception, microfluidics has demonstrated a tremendous potential to revolutionize the field of in vitro diagnostics (IVDs). Microfluidic IVD systems are believed to offer numerous advantages, such as portability, low cost, automation, and “sample-to-answer” capability, which could enable rapid, sensitive, and quantitative analyses of multiple targets by consuming minimal amounts of samples [1,2]. Especially, these microfluidic systems should be able to play vital roles in nucleic acid amplification tests (NATs) where the operation process is complicated and the prevention of contamination is a critical concern [3,4]. However, so far it is still uncommon to see microfluidic devices being routinely used in clinical diagnoses [5,6]. Why has this long-believed potential of microfluidics not been turned into reality yet?

To advance the microfluidics, researchers often borrowed ideas from the microelectronic industry, where the design and fabrication of electronic circuitries can be achieved by combining validated basic elements and processes [7,8,9]. Similarly, instead of developing isolated microfluidic systems, the implementation of a microfluidic platform, which comprises a combinable set of basic unit operations, is a much easier and less risky approach to translate in vitro diagnostic assays to the microchip format [9,10,11]. Indeed, in the last two decades, various microfluidic platforms have been successfully proposed for the integration and miniaturization of biochemical assays. According to the dominating liquid propulsion principles, the microﬂuidic platforms can be categorized to: capillary [12,13], mechanical driven [14], liquid or air pressure driven [15], centrifugal [11,16], electroosmotic [17], electrowetting [18], and acoustic systems [19]. A comprehensive analysis of the pros and cons of these microfluidic platforms can be found in a review reported by Mark et al. [10]. Undoubtedly, these platforms provide a full spectrum of tools for developing various microfluidic systems.

Unfortunately, when the IVD assays were miniaturized and integrated, some technical challenges associated with the specific requirements of clinical diagnosis arose and may be hurdles in the penetration of microfluidic systems into the IVD market [5]. The first challenge is the so-called “world-to-chip” interface, which includes two aspects: the reagent (sample) volume and the reagent (sample) type [20]. In clinical diagnosis, the volume of the sample needs to be large enough to have clinical relevance. For example, the accurate diagnosis of encephalitis requires a sensitivity down to 1 PFU/mL in cerebrospinal fluid [21]. As a result, a microfluidic device needs to handle at least 1 mL of sample, which is usually thousands of times higher than the volume of a typical microreactor (less than 1 μL) [22,23]. One solution is to attach enlarged reservoirs or tubes on microdevices to accommodate large-volume solutions [24,25]. However, these structures were only used as storage compartments and more complicated manipulations of large-volume reagents, such as mixing, have not been realized. Additionally, clinical samples may come in various forms: swabs, sputum, blood, urine, feces, etc. Since these samples are often viscous, prone to forming bubbles, and full of particles, the direct processing of raw samples was often excluded from an integrated microfluidic device [26,27]. Second, while more and more in vitro diagnoses have been translated into microfluidic formats, the integration of a complete bioassay into a single device remains as a formidable task that requires many rounds of trials and failures [28]. Although the modular design methods, such as Lego^®^-like [29,30] and plug-n-play [31] components, have been proposed, these technologies were inherently developed for quickly testing new ideas in a prototype way instead of product development [32]. Third, as the assays have become more and more complicated, the pursuit of throughput has not been stopped. It is not an easy task to simply array a microdevice considering the complicated microstructures and the “world-to-chip” interfaces that the device may have [28]. Furthermore, since an IVD system may be deployed to a variety of clinical settings, such as central laboratories, physicians’ offices, and bedsides, ideally, this system should be able to provide a throughput that can be adjusted according to the actual need at each run [6]. Overall, the current available microfluidic platforms fall short of addressing these challenges encountered in clinical diagnosis more or less and a more powerful design paradigm is highly desired.

In the current study, a “3D extensible” microfluidic design paradigm was developed to address the aforementioned critical issues in developing fully integrated IVD microsystems. Based on the classical elastic film microvalves actuated by pneumatics [33,34], we designed four basic function elements- check valve (in), check valve (out), double-check valve (in and out), and on–off valve, and two structure elements, chamber and compartment, fabricated using a consistent tape-based microfabrication technique. We proposed a “3D extensible” architecture to fulfill the needs of the function integration, the adaptive “world-to-chip” interface, and the adjustable throughput in the X, Y, and Z directions, respectively. To elucidate and verify this design paradigm, we developed a fully integrated loop-mediated isothermal amplification (iLAMP) microsystem with “sample-in-answer-out” capacity, adjustable throughput, and higher sensitivity compared with commercial kit. This example validated the feasibility of using this universal design paradigm to develop fully integrated and automated microfluidic systems in an easier, less risky, and more consistent manner.

## 2. Materials and Methods

### 2.1. Design Paradigm of the “3D Extensible” Microfluidic Systems

**Figure 1:**
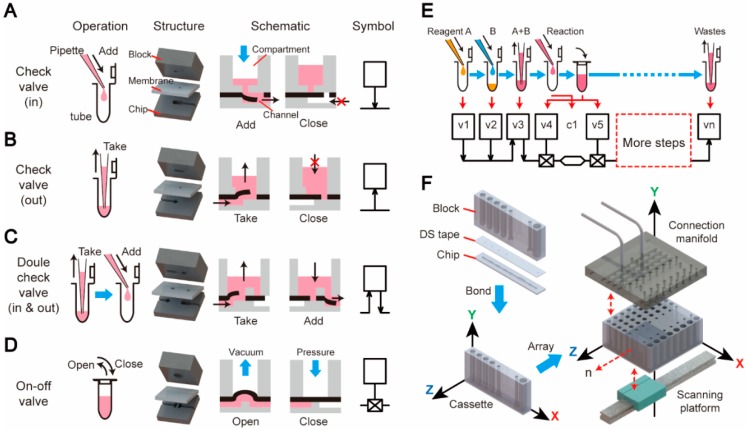
The basic elements and the architecture of the “3D extensible” microfluidic design paradigm. (**A**) A check valve (in), (**B**) a check valve (out), (**C**) a double-check valve, and (**D**) an on–off valve, were designed as four basic elements. (**E**) A schematic replicating a biochemical assay can be drawn by sequentially linking the symbols of the basic elements. (**F**) The linear arrangement of the basic elements can produce a cassette-like device, which shares the consistent three-layer structure as the basic elements: a 3D block, a piece of membrane (DS tape), and a 2D chip. The design of the microdevice is extensible in three directions: in the X direction, the combination of basic elements can be customized to achieve different functions; in the Y direction, the 3D block functions as the “world-to-chip” interface for liquid storage, fluid control, and signal detection; in the Z direction, the cassette can be arrayed to achieve an adjustable throughput according to the need at each run.

Similar to the digital logic gates (AND, OR, NOT, etc.) in a digital electronic circuit, the basic function elements in a microfluidic platform should mimic the most fundamental acts in a biochemical assay. As illustrated in Figure 1A–D, four basic acts in an assay were identified: adding a solution to a tube, taking a solution from a tube, taking and then adding a solution, and opening/closing a tube. These acts can be achieved by employing a series of derivative structures modified from the classical elastic membrane valve in which an elastic membrane is sandwiched between two chip layers [33,34]. As shown in the exploded views of Figure 1A–D, these sandwich structures consist of a 3D block, a membrane, and a thin chip. The block contains a compartment with a milliliter-scale volume, representing the macroscale section of the structure. The chip has microliter-scale channels and chambers fabricated on the upper side, representing the microscale section. As illustrated in Figure 1A, the act of “adding a solution to a tube” is realized using a check valve (in), in which a hole punched through the membrane has a flush contact with the bottom of the block. A pressure from the top compartment can bend the membrane to open the valve freely, but the reversed direction is stopped by the bottom of the block. By applying a pressure from the compartment, the solution stored in the compartment is driven into the microchannel on the chip, just like the act of “adding a solution from a pipette to a tube”. Similarly, a check valve (out) is employed to achieve the act of “taking a solution from a tube” (Figure 1B). The double-check valve is the combination of these two types of check valves for mimicking the act of “taking and then adding a solution” (Figure 1C). Finally, the on–off valve, which is the classical pneumatic microvalve, is used to shut off a channel connecting to a chamber (tube) in the chip, representing the closing or opening of a tube (Figure 1D). To make the design schematic more explicit, four symbols were assigned to these basic structures. The square in the symbol stands for the compartment in the block, the arrows indicate the flow directions of the check valves, and the horizontal lines are the channels located on the upper side of the chip. 

With these basic elements in hand, a complicated biochemical operation comprising a series of basic acts can be converted into a schematic diagram by linking the symbols of the selected elements via microchannels, just like drawing a schematic of an electric circuit using the electric components from a component library. For example, as shown in Figure 1E, reagent A and B are sequentially added into a tube and mixed. After the lid is closed, the reaction begins. Following these steps, more operations can be conducted to manipulate the products of the reaction, and finally, all the wastes are collected in a waste tube. Each act in this process can be represented by a basic element described above and all the elements are linked sequentially by the microchannels. The implementation of the linear arrangement of elements in the schematic can produce a slim, cassette-like device, which consists of three parts: a 3D block, double-sided tape, and a 2D chip, as illustrated in Figure 1F. This double-sided tape (DS tape) with an acrylic foam base (4910 VHB, 3M, Maplewood, MN, USA) was employed to bond the microdevice and to function as the elastic membrane in valves. In this cassette, the flow direction in the chip is defined as the X axle (the “function” direction), along which a series of basic elements are linked via the channels to perform a complete bioassay. The length of the chip along the X direction can be adjusted according to the integrated functions on the chip. In the vertical Y axle (the “interface” direction), the height of the block, which functions as the “world-to-chip” interface, can be adjusted according to the required volumes of the compartments in order to accommodate the samples and the reagents needed in the assays. All the control accesses are also applied to the device in the Y direction. Lastly, the cassette can be arrayed along the Z direction (the “throughput” direction) to achieve a higher throughput. The number of cassettes is adjustable to meet the throughput need of each run. A connection manifold can be pressed down to hold the device array in place and to provide all the pneumatic connections and external controls to the devices. This “3D extensible” device architecture can fulfill the specific demands of an IVD assay, including the function, the “world-to-chip” interface, and the throughput, in a flexible way.

### 2.2. Fabrication of “3D Extensible” Microfluidic Devices

The slim, cassette-like microdevice has three parts: a 3D block, a piece of patterned double-sided adhesive tape, and a planar chip. Both the block and the chip were made of poly(methyl methacrylate) (PMMA) using the conventional milling and drilling techniques. The non-adhesive patterning procedure of the DS tape is illustrated in Figure S1. Briefly, a pattern designed with AutoCAD (Version 2015, Autodesk, San Rafael, CA, USA) was carved onto a piece of release paper (CY9970, Yichuang Electric, Suzhou, China) using a flatbed cutting plotter (FC4500-50, Graphtec Corporation, Tokyo, Japan). Then, a piece of DS tape with its own release paper peeled off was covered with the patterned release paper as masks from both sides. Next, tris-HCl (pH = 8.0) was pipetted onto the exposed surfaces of the tape and incubated at 37 °C for 30 min to remove the adhesiveness. After the holes in check valves were manually punched and the masks were peeled off, the patterned DS tape was aligned to the block and the chip and pressed together with fingers to drive out any residual gas in the bonding interfaces. The assembled microdevice was kept at room temperature for at least 24 h before use in order to let the tape-bonding strength reach to its maximum. All the demo devices of unit operations were fabricated following this general procedure.

In the fabrication of the iLAMP microdevice, a piece of glass filter paper (GF/D, Whatman, GE Healthcare, Pittsburgh, PA, USA) modified with chitosan (molecular weight: 2000, Sigma-Aldrich, St. Louis, MO, USA) was embedded in the chamber for DNA capture and “in situ” amplification. The modification protocol of the glass filter paper can be found in our previous study [35]. Briefly, a piece of glass filter paper (47 mm diameter) with a thickness of 2 mm was first activated with oxygen plasma for 1 min, and then submerged in a chitosan solution (1% (w/v) in 1% acetic acid, pH = 5.0) followed by an overnight incubation on a tube roller. Then, the filter paper was washed with DI water three times and dried completely at 50 °C in a vacuum drying oven. The trapezoid-shaped filter with an area of 1 mm^2^ was punched off with a customized mental puncher and directly released into the end of the chamber on the chip. After that, a piece of adhesive PCR (polymerase chain reaction) plate foil (AB0626, Thermo Fisher, Waltham, MA, USA) patterned using the cutting plotter was carefully aligned and attached onto the upper side of the chip, covering the filter paper in the enclosed amplification chamber while leaving the microchannels open. This covered chip was pressed hardly with a manual hydraulic press (15-1-HT, GRIMCO, Paterson, NJ, USA) before being bonded with the block and the patterned DS tape.

### 2.3. Control and Detection Instrument

A control and detection instrument for the iLAMP microdevices was constructed with pneumatics for fluid manipulation, electronics for temperature control, and optics for fluorescence detection. Its core structure is shown in Figure S2. Up to eight microdevices can be put onto a Teflon stage, on which six pieces of ITO (indium tin oxides) glass (Meijingyuan Glass Technology, Foshan, China) were embedded side by side to form a heating zone with dimensions of 2.4 cm × 12 cm. The temperature control of the ITO heater was accomplished through a proportion/integrator/differentiator (PID) module, which used a thermocouple attached to the lower side of the first ITO glass for signal feedback. The microdevices were held in place by a custom-built connection manifold, which contained an array of pneumatic ports connected to a pneumatic control module. Within this module, two rotary vane pumps (G02-8, Gardner Denver Thomas, Fürstenfeldbruck, Germany) were employed to provide pressure (5.9 psi) and vacuum (−5.3 psi), respectively. Sixteen solenoid valves (LHLA1221111H, the Lee Company, Westbrook, CT, USA) were employed to switch between pressure, vacuum, and atmospheric pressure. Below the heating plate was the optical detection module. An optical box driven by a stepping motor scanned the amplification chambers of the chips through the ITO glasses. A 365-nm exciting beam from a LED (CREE 3535, Epileds, Tainan, Taiwan), first passed through a filter (et365/10×, Chroma, Brattleborro, VT, USA), was then reflected by a dichroic beam splitter (t455lpxt, Chroma, Brattleborro, VT, USA, and focused in the chamber by a convex lens (GCL-0101, Daheng Optics, Beijing, China). The excited fluorescence signal passed through the dichroic beam splitter and a filter (zet514/10×, Chroma, Brattleborro, VT, USA) was installed before a PMT (photomultiplier tubes, H9307, Hamamatsu, Shizuoka, Japan). A Raspberry Pi board (3B, Digi-Key, Shanghai, China) combined with a custom-build circuit board was developed for signal processing and controlling. 

### 2.4. DNA Extraction and Loop-Mediated Isothermal Amplification (LAMP) Reaction

Bacteriophage λ-DNA (Promega, Madison, WI, USA) was employed to examine the DNA capture efficiency of the iLAMP microdevice. After on-chip capture of λ-DNA, the chitosan-modified filter paper was taken out and placed into tubes for real-time PCR on a Bio-Rad iQ5 system (Bio-Rad, Hercules, CA, USA). In each tube, a 25 μL mixture was composed of 0.5 μL of forward/reverse primer (listed in Table S1), 12.5 μL of Power 2×SYBR real-time PCR premix (Thermo Fisher, Waltham, MA, USA), 11.5 μL of deionized (DI) water, and the filter paper. The thermal cycling protocol included an initial activation of Taq polymerases at 95 °C for 5 min, followed by 35 cycles of 95 °C for 30 s, 60 °C for 30 s, and 72 °C for 30 s, and a final extension step for 10 min at 72 °C.

The detection of *Chlamydia trachomatis* (CT) was realized by amplifying a specific sequence in its 7.5 kb cryptic plasmid. Each CT has 7~10 copies of this plasmid and the sequences of the template and LAMP primers are listed in Table S1. Swab samples were prepared as follows: one-microliter inactivated CT particles obtained from the CT Nucleic Acid Testing Kit (DAAN Gene, Guangzhou, China) was pipetted onto a urethral swab. After being air-dried, the swab tip was cut off and inserted into the device for the on-chip analysis. Since 250 μL of lysis buffer was employed to flush the swab, a total of 2500, 250, and 25 CT particles on the swabs can theoretically generate the lysates with concentrations of 10, 1, and 0.1 CT particles/μL, respectively. A 25 μL LAMP mixture contained 1.6 μM each of the inner primer (FIP and BIP), 0.2 μM each of the outer primer (F3 and B3), 0.4 μM of the loop primer (LF), 1× Isothermal Amplification Buffer (20 mM tris-HCl, 10 mM (NH_4_)_2_SO_4_, 50 mM KCl, 2 mM MgSO_4_, 0.1% Tween^®^ 20, pH 8.8@25 °C, NEB, Ipswich, MA, USA), 6.0 mM of MgSO_4_, 1.4 mM of dNTPs (Sangon Biotech, Shanghai, China), 0.5 M of betaine (Sigma-Aldrich, St. Louis, MO, USA), 0.15 mM of calcein (Coolaber, Beijing, China), 0.5 mM of MnCl_2_ (Tiandz, Beijing, China), 8 units of Bst 2.0 WarmStart^®^ DNA Polymerase (NEB, Ipswich, MA, USA), 6 μM Bovine serum albumin (BSA) (Sigma-Aldrich, St. Louis, MO, USA), and the template. The entire operation of the microdevice was performed automatically on a home-made instrument. The LAMP amplification graphs were plotted and outputted by the embedded system of the instrument, which also reported the threshold time based on predefined calibration curves.

## 3. Results and Discussion

### 3.1. Unit Operations of the Microfluidic Platform

**Figure 2:**
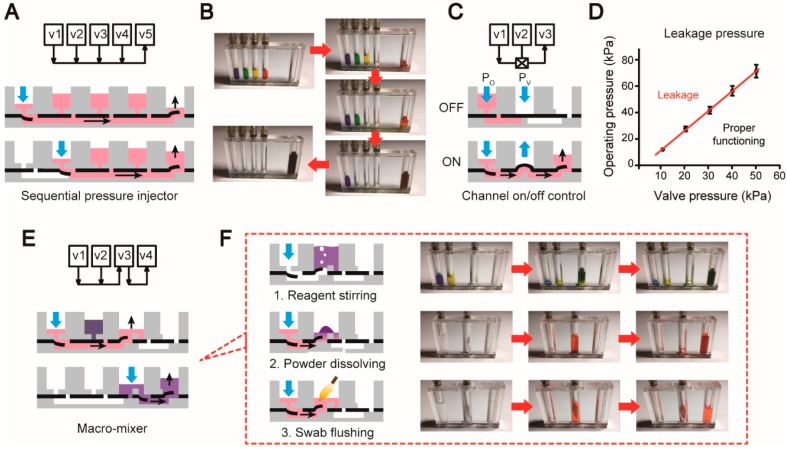
Unit operations of the microfluidic platform. The illustration (**A**) and validation (**B**) of the sequential pressure injector. The illustration (**C**) and quantification (**D**) of the channel on/off control (mean ± SD, n = 3). (**E**) The macro-mixer. (**F**) Different operations based on the macro-mixer: reagent stirring, powder dissolving, and swab flushing.

A microfluidic platform is usually required to provide a set of validated unit operations for fluid handlings, which can be combined and thereby realize application-specific assays on the platform. In our system, the aforementioned basic elements were used to design and construct a series of unit operations. First, multiple preloaded reagents are often sequentially loaded into the chip for downstream analysis. A sequential pressure injector was designed to accomplish this operation by linearly linking multiple check valves (in) (v1–v4) as illustrated in Figure 2A. Due to the one-way-flow property of these check valves, the reagents can be sequentially loaded into the chip by simply applying pressures to the valves one by one without the worry of mistakenly mixing reagents in the other compartments (Figure 2B and Video S1). In the end of this structure, a check valve (out) (v5) is employed as a waste reservoir to collect all the reagents from the chip. Second, during liquid transports, the fluid valving is often needed to control the flow path. As shown in Figure 2C, the fluidic valving can be easily achieved by adding an on–off valve (v2) between two check valves (v1 and v3). As shown in Figure 2D, for every P_V_, there is a critical P_O_ on the red fitted line that can burst out the valve. As a result, we need to make sure the working parameters are always below the red line to keep the valve close. To open the valve, a vacuum can be simply applied to the valve. The air pump we used in the instrument can provide a maximum Po of 59 kPa, which was sufficient to seal the amplification chamber during LAMP. As shown in Figure S3, the on–off valve could be properly closed as long as the pressure was below 59.0 kPa. After the pressure raised above this value, the liquid could be pushed through the pneumatic seal.

Fluid mixing is another indispensable unit operation for a microfluidic platform. Here we designed a macro-mixer containing two check valves (in) (v1 and v2), a double-check valve (v3), and a check valve (out) (v4). As illustrated in Figure 2E, the reagents stored in v1 and v2 are sequentially loaded into the v3 compartment, in which the reagents are mixed thoroughly by continuously bubbling. After that, the mixture is driven to v4 for the downstream analysis. We found this bubbling action could efficiently mix two reagents in less than 1 min. This structure can be easily modified to fit many unit operations that are often encountered in clinical diagnosis. As shown in Figure 2F, air can be blown into the compartment of the double-check valve, in which the floating bubbles disturb the liquid quickly to achieve the stirring effect. The same structure can also be used for dissolving freeze-dried powders: water is injected into the compartment to dissolve the powder freeze-dried in the compartment of the double-check valve followed by the bubbling vortex. Another function that can be achieved by this structure is the swab flushing. A swab, which is a common sampling mean in clinic diagnosis, is inserted into the compartment directly. Then, water or another reagent is pressed into the compartment and thoroughly flushes the swab by the bubbling vortex. All the unit operations described above form a “microfluidic component library” that can be assembled together to enable the design of any application-specific microfluidic system in a short turnaround time. This library can be expanded by incorporating more unit operations in the future.

### 3.2. Design Process of a Fully Integrated System for Pathogen Detection

To illustrate how to design an integrated microdevice using the “3D extensible” paradigm, we developed a fully integrated microsystem for nucleic acid-based pathogen detection. *Chlamydia trachomatis* (CT), the leading cause of sexually transmitted diseases (STDs) [36], was chosen as the target to test this microsystem. The development process of a fully integrated microsystem for pathogen detection based on the “3D extensible” design paradigm started with the determination of the biochemical assay that should be validated with the conventional off-chip operations in the first place. In the current study, first, *Chlamydia trachomatis* is usually sampled by urethral or vaginal swabs in clinical diagnosis. A thorough rinse of the sample swab in a lysis buffer could effectively release and lyse cells from the swab tip to the solution. Second, a chitosan-modified glass filter paper previously developed by our group was employed for the DNA extraction [35]. This filter paper-based method was chosen due to its over 90% DNA capture efficiency, the easy integration of a piece of filter within a microstructure, and the most attractive feature—“in situ” PCR capability, with which all the DNA captured on the filter paper can be directly used for amplification without elution. In the off-chip format, the filter paper with captured DNA was directly thrown into an Eppendorf tube for amplification. Likewise, a single microreactor should work for both the DNA extraction and the amplification on the device. Third, the amplification and detection of the extraction DNA on the filter paper was achieved using the loop-mediated isothermal amplification (LAMP) coupled with calcein-based real-time fluorescence detection. While LAMP has a poor capability of quantitating starting templates compared with that of real-time PCR, its rapid reaction, high sensitivity, and low requirements for the control and detection match the need of developing a rapid screening method for sexually-transmitted *C. trachomatis* infections in clinical diagnosis.

**Figure 3:**
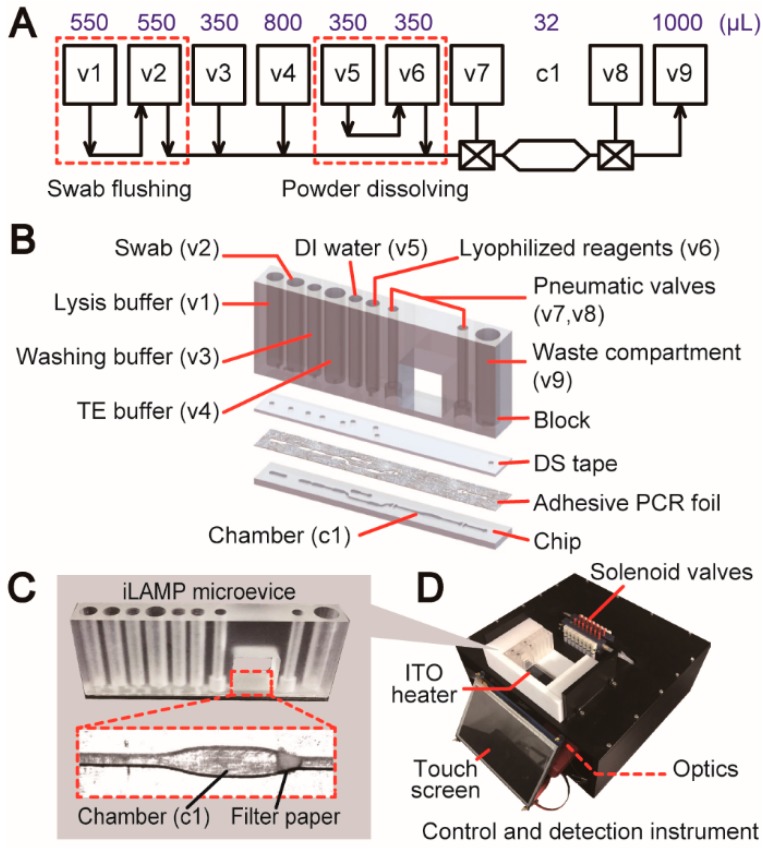
Design of the iLAMP system. (**A**) The schematic diagram of the iLAMP microdevice. v1–v9 represent the valve structures from the basic elements. c1 is the chamber on the chip. Volumes of each valve compartment and chamber are listed above corresponding structures. (**B**) The iLAMP device is comprised of a block, a piece of DS tape, and a chip covered with adhesive PCR foil. Reagents are prestored in valve compartments. (**C**) Photographs of the iLAMP device and the reaction chamber. A piece of chitosan-modified filter paper is embedded in the chamber for DNA capture and “in situ” LAMP. (**D**) Photograph of the control and detection instrument of the iLAMP device.

After the entire biochemical assay was finalized and validated, a schematic diagram was drawn using the unit operations and basic elements as building blocks to design a fully integrated LAMP (iLAMP) device that essentially replicated the entire procedure of the off-chip assay. As shown in Figure 3A, a unit operation of swab flushing (v1 and v2) was connected to a sequential pressure injector for DNA extraction and amplification. A powder dissolving unit (v5 and v6) was inserted into the design for dissolving lyophilized LAMP reagents with enzymes. A chamber (c1) with the embedded filter paper was employed as the reaction “tube”. Owing to the use of the chitosan-modified filter paper, both DNA capture and amplification were performed in this single chamber. Two on–off valves (v7 and v8) were employed to seal the chamber during amplification. Finally, a check valve (out) (v9) was designed in the end of the device to collect all the wastes driven through the chamber. Based on the device schematic, a microfluidic device can be further finalized and constructed by applying the rules of the “3D extensible” design paradigm. As illustrated in Figure 3B, this slim, cassette-like device consists of three major components: a 3D block containing compartments, a piece of DS tape, and a 2D chip. Three key issues need to be determined in the process from the schematic to the device: (i) the sizes of all the compartments in the block need to be determined according to the reagent volumes and the functions that are used in the assay; (ii) the pattern on the DS tape should be designed based on the types of the valves connected to the compartments; (iii) the microstructures on the upper side of the chip should be finalized based on the bottom horizontal lines in the schematic. In addition, a piece of chitosan-modified filter paper was embedded in the end of the chamber (c1) to enable DNA capture (Figure 3C). A piece of patterned non-transparent adhesive PCR plate foil was attached onto the upper side of the chip before bonding, providing a uniform fluorescence background for detection and a biocompatible surface for more efficient amplification. The cassette-like iLAMP microdevice has dimensions of 76.5 mm × 10 mm × 32 mm and the detailed design can be found in Figure S4. The microdevice was operated on the home-made instrument which was developed according to the needs of the assay (Figure 3D). Up to eight microdevices could be loaded on the instrument side by side in an array and the number of the devices can be flexibly adjusted according to the need of each run.

### 3.3. Operation of the iLAMP Microsystem

**Figure 4:**
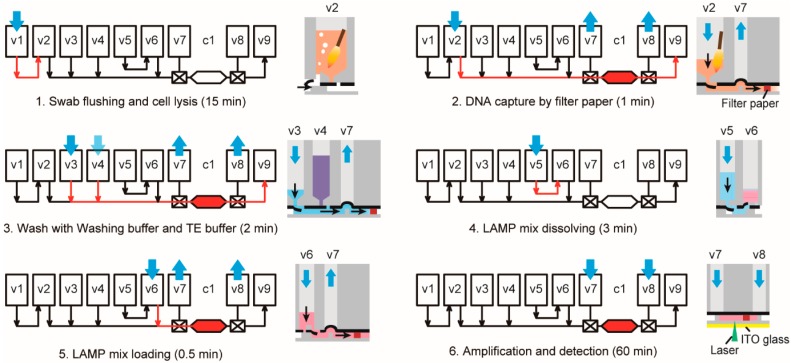
Operation of the iLAMP microsystem. The total analytical time was about 82 min, including 22 min for DNA extraction and 60 min for LAMP.

As demonstrated in Figure 4 and Video S2, after the swab insertion and the device loading, the rest of the procedure of the *C. trachomatis* detection could be automatically conducted under the control of the instrument without any manual interventions. Briefly, the swab tip was first inserted into the sample compartment (v2) of the device which was then sealed by the connection manifold on the instrument. Lysis buffer (v1) was injected into the swab compartment and air was continuously blown for 15 min to flush the swab by the bubbling vortex. The lysate was then driven through the chamber (c1) containing the filter paper, by which DNA was captured. Then, the washing buffer (v3) and the TE solution (v4) were sequentially injected through the paper to remove the residual lysis buffer and to neutralize the pH in the chamber. The LAMP mix (v6), which was dissolved by adding DI water (v5), was injected slowly to fill the reaction chamber without introducing any air bubbles. Finally, the valves (v7 and v8) at both ends of the chamber were closed and the chamber was heated by the ITO heater underneath the device. Temperature calibration showed that the chamber was heated to 65 °C in 5 min and maintained for 60 min (Figure S5). Real-time fluorescence signals were recorded by the scanning PMT in the detection instrument. 

### 3.4. Evaluation of Analytical Steps

**Figure 5:**
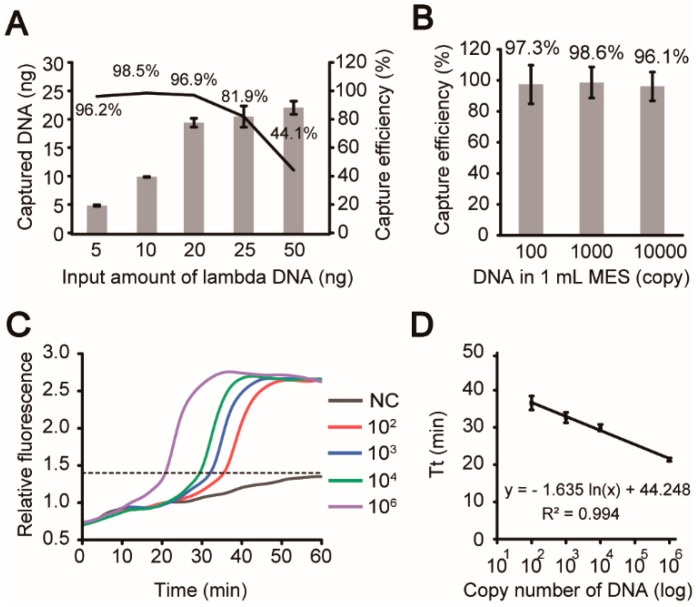
Characterization and evaluation of the iLAMP system. Evaluation of DNA capture by the chitosan-modified filter paper in the iLAMP device using λ-DNA samples. (**A**) The average DNA capture efficiencies were above 96% when input was between 5–20 ng (mean ± SD, n = 3). (**B**) The average capture efficiencies were above 96% when 10,000, 1000, or even 100 copies of λ-DNA were diluted in 1 mL MES (mean ± SD, n = 3). (**C**) Validation of on-chip amplifications of 0, 10^2^, 10^3^, 10^4^, and 10^6^ copies of templates. The experiments were repeated three times and only one set of results were shown here. (**D**) The fitted curve between the log of the template copy number and the threshold time (Tt) in the fluorescence graphs (mean ± SD, R^2^ = 0.994, n = 3).

The DNA capture by the chitosan-modified filter paper was first verified on the device. Previously, we had proved this filter paper could provide a high DNA capture efficiency [35,37]. However, in the current system, since this filter was embedded into the chamber in a lateral flow format, its performance should be carefully optimized and tested. First, different amounts of λ-DNA prepared in 1 mL MES (2-(N-morpholino) ethanesulfonic acid) solution (pH = 5.0) were injected into the chamber at a flow rate of 1 mL/min using a syringe pump, followed by washes with 50 μL 1% SDS (sodium dodecyl sulfate) and 200 μL 1× TE buffer. After that, the filter paper was taken out from the chip and transferred into a PCR tube for real-time PCR quantitation of captured DNA on the filter. Figure 5A illustrated that the capture efficiencies were kept above 96% when the input DNA was in the range of 5–20 ng, and the efficiencies declined gradually with the input amounts increased to 25 and 50 ng due to the saturation of the filter paper. Therefore, we estimated the DNA capture capacity of our system is in the range of 20 to 25 ng. When the template amount was further reduced to 10,000, 1000, and even 100 copies of λ-DNA diluted in 1 mL MES, the average capture efficiencies were still higher than 96% (Figure 5B). Such an extraordinary capture efficiency with highly diluted DNA resulted from the sufficient interactions between DNA and the filter paper in the lateral flow mode. After the verification of the on-chip DNA capture, we next tested the on-chip isothermal amplification and detection of a specific sequence in the cryptic plasmid of *Chlamydia trachomatis*. A series of 15 μL LAMP mixtures, containing 10^2^, 10^3^, 10^4^, and 10^6^ copies of template along with DI water as negative controls, were injected into the chambers for LAMP tests at 65 °C for 60 min. The typical real-time fluorescence graphs were shown in Figure 5C and the average threshold time (Tt) calculated from three repeats (Figure S6) was plotted as a function of the log of the template copy number in Figure 5D. The linear fit with an R^2^ of 0.994 confirmed the reliable LAMP reactions and the fluorescence detections on the device.

### 3.5. “Sample-In-Answer-Out” Analyses in the iLAMP System

**Figure 6:**
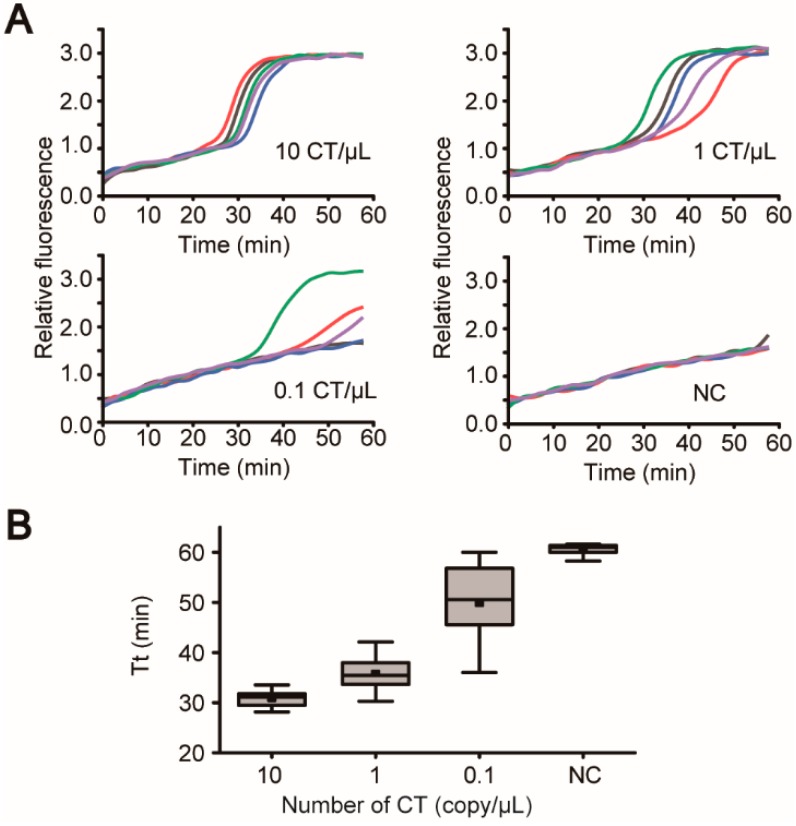
“Sample-in-answer-out” analyses of mock clinical samples using the iLAMP system. (**A**) Swab samples producing 10, 1, and 0.1 CT particles/μL in the lysis buffer tested on the iLAMP microsystem. In each group, five microdevices were operated simultaneously on the instrument. Only 3 in 5 tests successfully amplified the targets in the 0.1 CT/μL group, indicating the system had reached its limit of detection. (**B**) The boxplot between the input numbers of CT particles and the amplification threshold times in the “sample-in-answer-out” analyses (n = 5).

Following the verification of each analytical step independently, the entire assay was tested on the device to prove the “sample-in-answer-out” capability of the iLAMP system. Swabs containing 2500, 250, and 25 CT particles were employed as the mock clinical samples. At each concentration, five microdevices were loaded onto the instrument and tested simultaneously by following the procedure described above. The real-time fluorescence graphs at the concentrations of 10 and 1 CT/μL demonstrated steep rises of the baseline fluorescence signals, indicating the successful amplifications of the target sequences of the CT particles (Figure 6A). By contrast, the steep rises of the signals were either delayed or disappeared at the concentration of 0.1 CT/μL, suggesting that the system had reached its limit of detection (LOD). The threshold times extracted from these graphs were also plotted as a function of the concentration of CT particles. Figure 6B showed that a negative correlation was established between the sample concentration and the Tt. The LOD of our system was determined to be 1 CT particles/μL, which was 10 times higher than that of the commercial kit (DAAN Gene, 10 CT particles/μL). In addition, the turnaround time of the iLAMP is about 82 min, which is slightly faster than that of the conventional method using the DAAN kit (~90 min). Since LAMP is an isothermal amplification method, it is prone to non-specific amplifications. In our study, when the amplification time was set to longer than 60 min, there were more chances to get false positive results (Figure 6A). But by limiting the time to 60 min, reliable results could be provided by the iLAMP microsystem.

The iLAMP system proved the excellent design capability of the “3D extensible” paradigm. Along the length direction of the microdevice, the functions were realized by linking a series of proved unit operations from the “microfluidic component library”, and the same method can be applied to develop more IVD microsystems after further improvements, including reagents mixing and quantification. The extensibility in the height direction of the block provided the capacity for swab handling and reagents storage, thereby making a fully integrated and fully enclosed microdevice for “sample-in-answer-out” pathogen detection. This cassette-like microdevice can be arrayed along its width direction to achieve an adjustable throughput on a control and detection instrument. The high sensitivity proved by repeated experiments could be mainly attributed to the enrichment of nucleic acid by the filter paper and the “in situ” amplification. In the shelf time test, the microdevices with preloaded reagents were stored at −20 °C and the on-chip amplification was still successful after 40 days, demonstrating the good biocompatibility of the material as well as the reliability of the structures. In future, the mass production of the microdevice could be realized by plastic injection molding coupled with the convenient tape bonding, providing a powerful and cost-efficient alternative for pathogen detection in the IVD market. 

## 4. Conclusions

Our “3D extensible” design paradigm is a universal microfluidic platform specially developed for use in clinical diagnosis. As the proofs of concepts, here we successfully developed an iLAMP system for pathogen detection. The iLAMP system possessed an excellent “world-to-chip” interface for liquid exchange between micro- and macro-scale, reagent storage, and convenient interaction with external peripherals, a compact integration for the “sample-in-answer-out” operations, and an adjustable throughput to meet the uncertainty in the practical application. Our study clearly demonstrated the central role that the “3D extensible” design paradigm may play in the development of microfluidic systems for IVD. In addition, although we focused our efforts to the nucleic acid testing in the current study due to the complexity of the NATs, we believe other types of clinical diagnosis, such as immunoassays, can all be realized using the “3D extensible” design method. We admit that our design paradigm still requires further development, such as the mass production and the microfluidic component library. Nevertheless, our study successfully provides a universal design paradigm that researchers can adopt to quickly develop integrated microsystems for various IVD assays in the future.

## Supplementary Materials

The following are available online at https://www.mdpi.com/2072-666X/10/12/873/s1, Table S1: Primers and ordered sequences, Figure S1: Patterning procedure of the tape, Figure S2: Core structure of the iLAMP instrument, Figure S3: Quantitative characterization of the pneumatic microvalves, Figure S4: Drawings of the iLAMP microdevice, Figure S5: Temperature calibration of the iLAMP instrument, Figure S6: Validation of on-chip amplification, Video S1: The demo of unit operations, Video S2: The working process of the iLAMP microdevice.

## References

[1] Whitesides G.M. (2006). The origins and the future of microfluidics. Nature.

[2] Sackmann E.K., Fulton A.L., Beebe D.J. (2014). The present and future role of microfluidics in biomedical research. Nature.

[3] Yeo L.Y., Chang H.C., Chan P.P., Friend J.R. (2011). Microfluidic devices for bioapplications. Small.

[4] Nayak S., Blumenfeld N.R., Laksanasopin T., Sia S.K. (2017). Point-of-care diagnostics: Recent developments in a connected age. Anal. Chem..

[5] Li P. (2012). Microfluidics for IVD: In pursuit of the holy grail. J. Bioeng. Biomed. Sci..

[6] Chin C.D., Linder V., Sia S.K. (2012). Commercialization of microfluidic point-of-care diagnostic devices. Lab Chip.

[7] Ahn C.H., Choi J.W., Beaucage G., Nevin J., Lee J.B., Puntambekar A., Lee R.J.Y. (2004). Disposable smart lab on a chip for point-of-care clinical diagnostics. Proc. IEEE.

[8] Bhargava K.C., Thompson B., Malmstadt N. (2014). Discrete elements for 3D microfluidics. Proc. Natl. Acad. Sci. USA.

[9] Haeberle S., Zengerle R. (2007). Microfluidic platforms for lab-on-a-chip applications. Lab Chip.

[10] Mark D., Haeberle S., Roth G., von Stetten F., Zengerle R. (2010). Microfluidic lab-on-a-chip platforms: requirements, characteristics and applications. Chem. Soc. Rev..

[11] Strohmeier O., Keller M., Schwemmer F., Zehnle S., Mark D., von Stetten F., Zengerle R., Paust N. (2015). Centrifugal microfluidic platforms: advanced unit operations and applications. Chem. Soc. Rev..

[12] Martinez A.W., Phillips S.T., Whitesides G.M., Carrilho E. (2010). Diagnostics for the developing world: Microfluidic paper-based analytical devices. Anal. Chem..

[13] Yetisen A.K., Akram M.S., Lowe C.R. (2013). Paper-based microfluidic point-of-care diagnostic devices. Lab Chip.

[14] Yang H., Chen Z., Cao X., Li Z., Stavrakis S., Choo J., deMello A.J., Howes P.D., He N. (2018). A sample-in-digital-answer-out system for rapid detection and quantitation of infectious pathogens in bodily fluids. Anal. Bioanal. Chem..

[15] Easley C.J., Karlinsey J.M., Bienvenue J.M., Legendre L.A., Roper M.G., Feldman S.H., Hughes M.A., Hewlett E.L., Merkel T.J., Ferrance J.P. (2006). A fully integrated microfluidic genetic analysis system with sample-in-answer-out capability. Proc. Natl. Acad. Sci. USA.

[16] Gorkin R., Park J., Siegrist J., Amasia M., Lee B.S., Park J.M., Kim J., Kim H., Madou M., Cho Y.K. (2010). Centrifugal microfluidics for biomedical applications. Lab Chip.

[17] Snyder J.L., Getpreecharsawas J., Fang D.Z., Gaborski T.R., Striemer C.C., Fauchet P.M., Borkholder D.A., McGrath J.L. (2013). High-performance, low-voltage electroosmotic pumps with molecularly thin silicon nanomembranes. Proc. Natl. Acad. Sci. USA.

[18] Abdelgawad M., Wheeler A.R. (2009). The digital revolution: A new paradigm for microfluidics. Adv. Mater..

[19] Voiculescu I., Nordin A.N. (2012). Acoustic wave based MEMS devices for biosensing applications. Biosens. Bioelectron..

[20] Kim J., Johnson M., Hill P., Gale B.K. (2009). Microfluidic sample preparation: Cell lysis and nucleic acid purification. Integr. Biol..

[21] Parida M.M., Santhosh S.R., Dash P.K., Tripathi N.K., Saxena P., Ambuj S., Sahni A.K., Lakshmana-Rao P.V., Morita K. (2006). Development and evaluation of reverse transcription-loop-mediated isothermal amplification assay for rapid and real-time detection of Japanese encephalitis virus. J. Clin. Microbiol..

[22] Campos C.D.M., Gamage S.S.T., Jackson J.M., Witek M.A., Park D.S., Murphy M.C., Godwin A.K., Soper S.A. (2018). Microfluidic-based solid phase extraction of cell free DNA. Lab Chip.

[23] Xu G., Hsieh T.M., Lee D.Y., Ali E.M., Xie H., Looi X.L., Koay E.S., Li M.H., Ying J.Y. (2010). A self-contained all-in-one cartridge for sample preparation and real-time PCR in rapid influenza diagnosis. Lab Chip.

[24] Nguyen H.V., Nguyen V.D., Lee E.Y., Seo T.S. (2019). Point-of-care genetic analysis for multiplex pathogenic bacteria on a fully integrated centrifugal microdevice with a large-volume sample. Biosens. Bioelectron..

[25] Hoffmann J., Mark D., Lutz S., Zengerle R., von Stetten F. (2010). Pre-storage of liquid reagents in glass ampoules for DNA extraction on a fully integrated lab-on-a-chip cartridge. Lab Chip.

[26] Ferguson B.S., Buchsbaum S.F., Wu T.T., Hsieh K., Xiao Y., Sun R., Soh H.T. (2011). Genetic analysis of H1N1 influenza virus from throat swab samples in a microfluidic system for point-of-care diagnostics. J. Am. Chem. Soc..

[27] Sun Y., Haglund T.A., Rogers A.J., Ghanim A.F., Sethu P. (2018). Review: Microfluidics technologies for blood-based cancer liquid biopsies. Anal. Chim. Acta.

[28] Culbertson C.T., Mickleburgh T.G., Stewart-James S.A., Sellens K.A., Pressnall M. (2014). Micro total analysis systems: Fundamental advances and biological applications. Anal. Chem..

[29] Hsieh Y.-F., Yang A.-S., Chen J.-W., Liao S.-K., Su T.-W., Yeh S.-H., Chen P.-J., Chen P.-H. (2014). A Lego®-like swappable fluidic module for bio-chem applications. Sens. Actuators B.

[30] Vittayarukskul K., Lee A.P. (2017). A truly Lego (R)-like modular microfluidics platform. J. Micromech. Microeng..

[31] Meng Z.-J., Wang W., Liang X., Zheng W.-C., Deng N.-N., Xie R., Ju X.-J., Liu Z., Chu L.-Y. (2015). Plug-n-play microfluidic systems from flexible assembly of glass-based flow-control modules. Lab Chip.

[32] Yuen P.K. (2016). A reconfigurable stick-n-play modular microfluidic system using magnetic interconnects. Lab Chip.

[33] Zhang W., Lin S., Wang C., Hu J., Li C., Zhuang Z., Zhou Y., Mathies R.A., Yang C.J. (2009). PMMA/PDMS valves and pumps for disposable microfluidics. Lab Chip.

[34] Ogilvie I.R., Sieben V.J., Cortese B., Mowlem M.C., Morgan H. (2011). Chemically resistant microfluidic valves from Viton® membranes bonded to COC and PMMA. Lab Chip.

[35] Gan W., Gu Y., Han J., Li C.X., Sun J., Liu P. (2017). Chitosan-modified filter paper for nucleic acid extraction and “in situ PCR” on a thermoplastic microchip. Anal. Chem..

[36] Meyer T. (2016). Diagnostic procedures to detect Chlamydia trachomatis infections. Microorganisms.

[37] Hui J., Gu Y., Zhu Y., Chen Y., Guo S.J., Tao S.C., Zhang Y., Liu P. (2018). Multiplex sample-to-answer detection of bacteria using a pipette-actuated capillary array comb with integrated DNA extraction, isothermal amplification, and smartphone detection. Lab Chip.

