# Supplementary material for: A Fully Integrated In Vitro Diagnostic Microsystem for Pathogen Detection Developed Using a “3D Extensible” Microfluidic Design Paradigm
Source: Micromachines (Basel). 2019 Dec 12;10(12):873. doi: 10.3390/mi10120873 (PMC6953088; doi:10.3390/mi10120873)
Supplement: Supplementary file 1 [file micromachines-10-00873-s001.zip › micromachines-664151 Supplementary Materials/micromachines-664151 Supplementary Tables and Figures.pdf]

micromachines

Supplementary Materials: A fully Integrated in vitro Diagnostic Microsystem for Pathogen Detection Developed Using a "3D Extensible" Microfluidic Design Paradigm

Zhi Geng [1], Yin Gu [1,2], Shanglin Li [1,2], Baobao Lin [1] and Peng Liu [1,*]

1 Department of Biomedical Engineering, School of Medicine, Tsinghua University, Beijing 100084, China; gengz15@mails.tsinghua.edu.cn (Z.G.); y-gu13@tsinghua.org.cn (Y.G.); li-sl14@tsinghua.org.cn (S.L.); lbb17@mails.tsinghua.edu.cn (B.L.)
2 FengteBio Corporation, Beijing 100079, China;
* Correspondence: pliu@tsinghua.edu.cn; Tel.: +86-10-6279-8732

Received: 25 November 2019; Accepted: 10 December 2019; Published: date

Table S1. Primers and ordered sequences (5′ to 3′).

Primer Species	Ordered Sequences
λ-DNA, PCR forward primer	CAAGCTTTGCCACACCACGGTATT
λ-DNA, PCR reverse primer	TAAGCACGAACTCAGCCAGAACGA
CT, LAMP inner primer, FIP	ACGTCTTTGTTTCTAGATGAAGGAAAGTATGTGGAATGT CGAACT
CT, LAMP inner primer, BIP	AGTCTGATTCAGAGAAGAATCGCCGGAACACATGATGC GAAGT
CT, LAMP outer primer, F3	GGCGATTTAAAAACCAAGGTC
CT, LAMP outer primer, B3	AGGAGGACAAAGAAACTCC
CT, LAMP loop primer, LF	CCAACACCCTTATCGCCGA
CT plasmid sequence	TCTCAGCAACTTTGAATTCTGAGGAAAGTCAGAGTTTG GATCAATTATTTTTATCAGAGTCCCAAAACTATTCGGAT GAAGAATTTTATCAAGAAGACATCCTAGCGGTAAAACT GCTTACTGGTCAGATAAAATCCATACAGAAGCAACACG TACTTCTTTTAGGAGAAAAAATCTATAATGCTAGAAAAA TCCTGAGTAAGGATCACTTCTCCTCAACAACTTTTTCAT CTTGGATAGAGTTAGTTTTTAGAACTAAGTCTTCTGCTTA CAATGCTCTTGCATATTACGAGCTTTTTATAAACCTCCCC AACCAAACTCTACAAAAAGAGTTTCAATCGATCCCCTA TAAATCCGCATATATTTTGGCCGCTAGAAAAGGCGATTT AAAAACCAAGGTCGATGTGATAGGGAAAGTATGTGGA ATGTCGAACTCATCGGCGATAAGGGTGTTGGATCAATTT CTTCCTTCATCTAGAAACAAAGACGTTAGAGAAACGAT AGATAAGTCTGATTCAGAGAAGAATCGCCAATTATCTG ATTTCTTAATAGAGATACTTCGCATCATGTGTTCCGGAGT TTCTTTGTCCTCCTATAACGAAAATCTTCTACAACAGCTT TTTGAACTTTTTAAGCAAAAGAGCTGATCCTCCGTCAGC TCATATATATATCTATTATATATATATATTTAGGGATTTGAT TTTACGAGAGAG

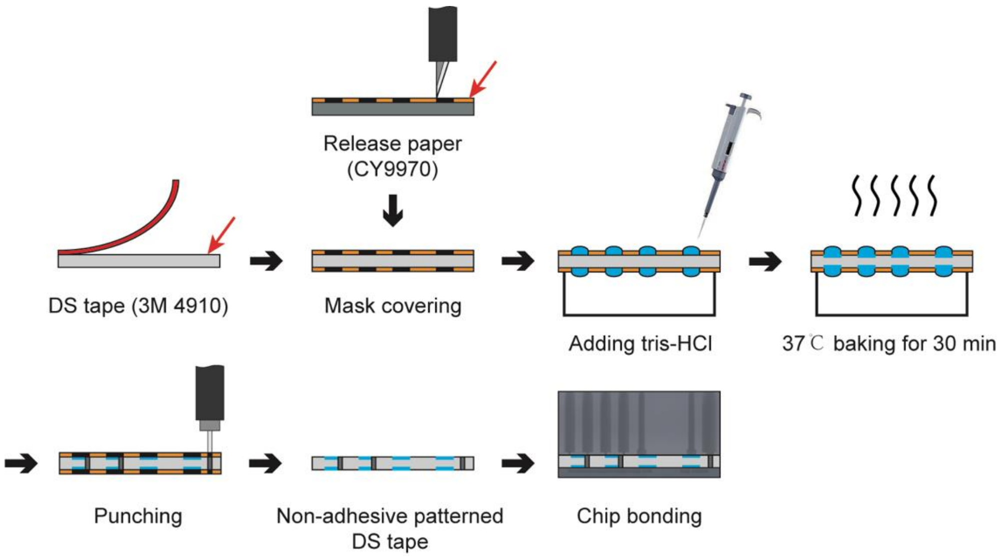

Figure S1. Patterning procedure of the tape. The double-sided adhesive (DS) tape (3M 4910) was covered on both sides by patterned release paper. Then, tris-HCl was pipetted onto the exposed surfaces of the tape followed by incubation at 37 °C for 30 min for removing the adhesiveness. After the holes for check valves were punched and the masks were peeled off, the patterned DS tape was employed for device bonding.

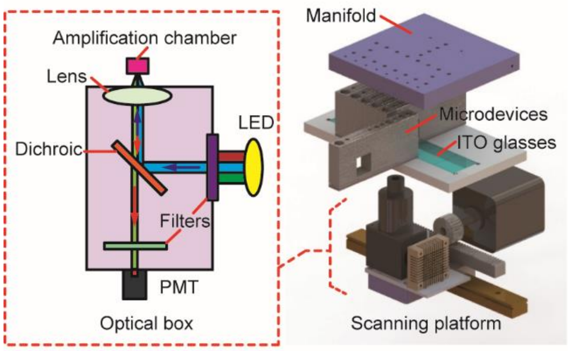

Figure S2. Core structure of the iLAMP instrument. The amplification chambers of iLAMP microdevices were heated by ITO glasses and a connection manifold served as the adapter between microdevices and air pumps. An optical box containing a PMT, a LED, lens, and filters, was driven by a stepping motor to scan the fluorescence of the amplification chambers.

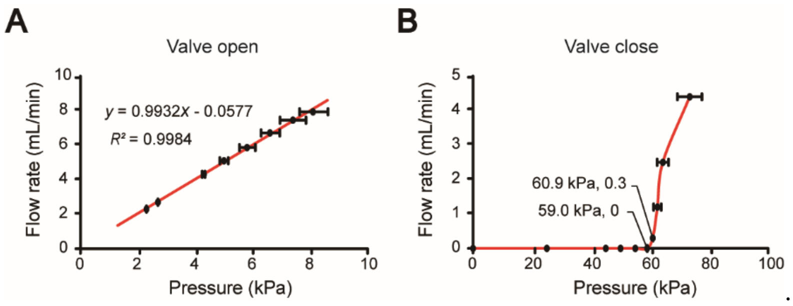

Figure S3. Quantitative characterization of the pneumatic microvalves. The inlet of the device in Figure 2C was connected via PTFE tubing to a 50-mL syringe filled with water and installed on a syringe pump and a manometer was also connected to the syringe via a Y-adapter to measure the pressure. For measuring the flow rate, the outlet of the chip was connected via PTFE tubing to a flowmeter and a water tank in the end. (**A**) When the on-off valve (v2) was open, the flow rate was proportional to the working pressure and the coefficient reflected the flow resistance (mean ± SD, n = 3). (**B**) When a pressure (Pv = 5.9 psi) was applied to the valve (v2) using a rotary vane pump, the valve (v2) could be properly closed as long as the pressure (PO) below 59.0 kPa. After the pressure (Po) raised above this value, the liquid could be pushed through the pneumatic seal (mean ± SD, n = 3).

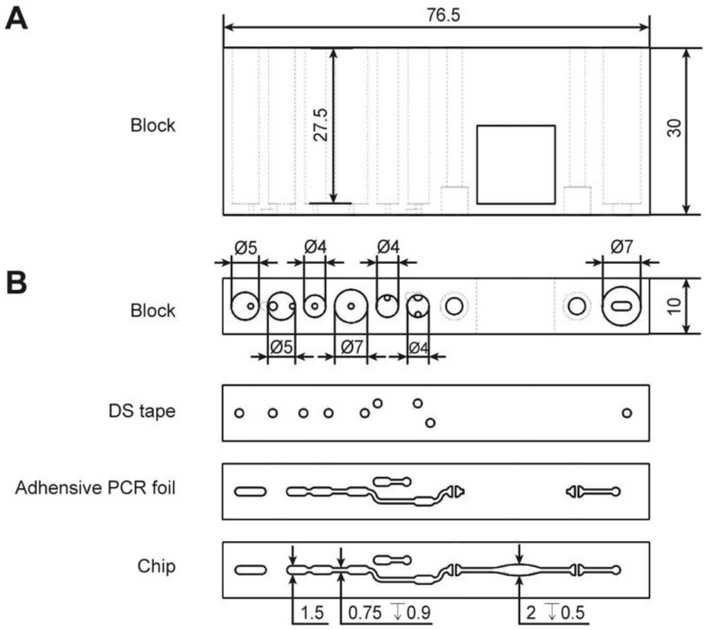

Figure S4. Drawings of the iLAMP microdevice. All the dimensions are shown in millimeters. (**A**) Side view of the block. The cavity above the amplification chamber is used for thermal insulation, leading to an increased heating rate. (**B**) Top views of different components of the iLAMP microdevice.

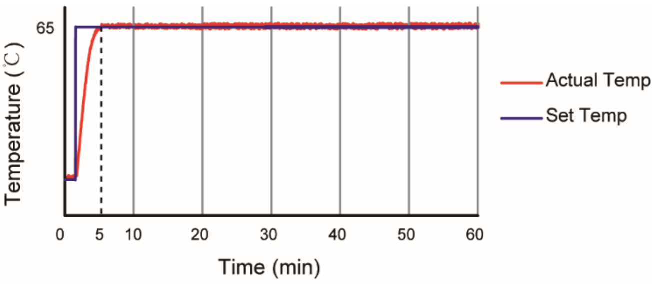

Figure S5. Temperature calibration of the iLAMP instrument. The set temperature (**blue**) was calibrated according to the actual temperature in the amplification chamber of the iLAMP microdevice (**red**). The chamber was heated to 65 °C in 5 min and maintained for 60 min.

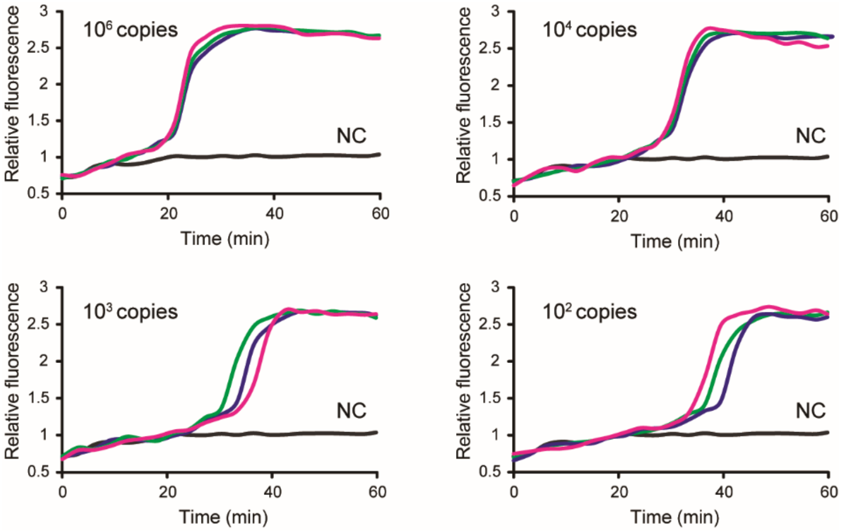

Figure S6. Validation of on-chip amplifications of 10^6, 10^4, 10^3, and 10^2 copies of templates. The fluorescence graphs showed that the positive groups had steep signal rises from the baseline. The experiments in each group were repeated three times.
